# Head Size in Phelan–McDermid Syndrome: A Literature Review and Pooled Analysis of 198 Patients Identifies Candidate Genes on 22q13

**DOI:** 10.3390/genes14030540

**Published:** 2023-02-21

**Authors:** Sara M. Sarasua, Jane M. DeLuca, Curtis Rogers, Katy Phelan, Lior Rennert, Kara E. Powder, Katherine Weisensee, Luigi Boccuto

**Affiliations:** 1 Healthcare Genetics and Genomics Program, Clemson University School of Nursing, Clemson, SC 29634, USA; 2 Greenwood Genetic Center, Greenville, SC 29605, USA; 3 Florida Cancer Specialists & Research Institute, Fort Myers, FL 33908, USA; 4 Department of Public Health Sciences, Clemson University, Clemson, SC 29634, USA; 5 Department of Biological Sciences, Clemson University, Clemson, SC 29634, USA; 6 Department of Sociology, Anthropology and Criminal Justice, Clemson University, Clemson, SC 29634, USA

**Keywords:** Phelan–McDermid syndrome, 22q13 deletion, macrocephaly, microcephaly, head circumference, genotype-phenotype correlation

## Abstract

Phelan–McDermid syndrome (PMS) is a multisystem disorder that is associated with deletions of the 22q13 genomic region or pathogenic variants in the *SHANK3* gene. Notable features include developmental issues, absent or delayed speech, neonatal hypotonia, seizures, autism or autistic traits, gastrointestinal problems, renal abnormalities, dolichocephaly, and both macro- and microcephaly. Assessment of the genetic factors that are responsible for abnormal head size in PMS has been hampered by small sample sizes as well as a lack of attention to these features. Therefore, this study was conducted to investigate the relationship between head size and genes on chromosome 22q13. A review of the literature was conducted to identify published cases of 22q13 deletions with information on head size to conduct a pooled association analysis. Across 56 studies, we identified 198 cases of PMS with defined deletion sizes and head size information. A total of 33 subjects (17%) had macrocephaly, 26 (13%) had microcephaly, and 139 (70%) were normocephalic. Individuals with macrocephaly had significantly larger genomic deletions than those with microcephaly or normocephaly (*p* < 0.0001). A genomic region on 22q13.31 was found to be significantly associated with macrocephaly with *CELSR1*, *GRAMD4*, and *TBCD122* suggested as candidate genes. Investigation of these genes will aid the understanding of head and brain development.

## 1. Introduction

Head circumference, or occipitofrontal circumference (OFC), is a standard measure in clinical practice, systematically collected since birth, and relatively easy to obtain and track over a subject’s growth curve. Measurement of the head size provides valuable information on the growth and development of the cranium and, indirectly, the brain. The terms macrocephaly and microcephaly are generally used to indicate head size measures above the 97th and below the 3rd percentile, respectively, for a given age. They can be present either isolated or accompanied by craniofacial dysmorphic traits and/or brain abnormalities. In several microdeletion syndromes, such as Cri-du-chat syndrome (5p deletion), or Williams syndrome (7q11.23 deletion), the loss of several genes likely leads to the disruption of molecular pathways regulating growth and development and thus is associated with microcephaly, although some exceptions include microdeletion syndromes presenting with normocephaly or even macrocephaly, such as Sotos syndrome (5q35 deletion). Thus, clinical assessment of head size is instrumental in investigating the functional impact of haploinsufficiency of deleted genes and the affected pathways in brain and skull development.

This study focuses on abnormal head size in subjects with Phelan–McDermid syndrome (PMS) caused by chromosomal deletion of the 22q13 region to investigate the correlation between this clinical feature and the haploinsufficiency of *SHANK3* and other 22q13 genes. Typical PMS features include developmental delay, neonatal hypotonia, motor impairment, delayed or absent speech, seizures, autistic traits, sleep disorders, and gastrointestinal and renal issues [1,2,3]. The phenotype of this syndrome is characterized by remarkable clinical variability involving, among other features, a spectrum of head size presentations, ranging from micro- to macrocephaly [4]. The genetic abnormalities leading to PMS are also relatively heterogeneous, ranging from pathogenic variants of the *SHANK3* gene to terminal deletions of the 22q13 region spanning over 9 Mb, and including chromosomal rearrangements such as ring chromosome 22, unbalanced translocations, and interstitial deletions [1,5,6,7,8]. Due to such clinical and genetic variability, a distinction has been recently proposed between cases including *SHANK3* variants or *SHANK3* haploinsufficiency due to 22q13 rearrangements (PMS *SHANK3*-related) and cases with preserved *SHANK3* (PMS *SHANK3*-unrelated) [9]. Historically, assessment of the prevalence of head size abnormalities in PMS has been hampered by small study cohorts or lack of systematic examination; moreover, early reports of subjects with PMS emphasized how growth parameters showed normal to accelerated patterns, including OFC, which was a relatively unusual feature for a microdeletion syndrome at that time. Moreover, a relatively common dysmorphic trait in PMS is dolichocephaly, reported in over 25% of cases [1], which can often lead to biased reports of macrocephaly. However, in recent years more refined genetic tests have allowed increasing the diagnostic yield for PMS and extending the phenotypic spectrum by including numerous cases with normal to decreased head size [1,4]. The variability of this trait and the novel classification of PMS subgroups call for a more accurate assessment of the prevalence of macro- and microcephaly in PMS, with particular attention to possible associations with deletion size, location, and genomic loci on 22q13. We hypothesize that larger deletions, indicative of a loss of additional haploinsufficient genes, may be associated with a higher risk of abnormal head size and possibly brain abnormalities. Therefore, exploring the genotype-phenotype correlation between macro/microcephaly and chromosomal deletions in PMS may highlight the contribution of 22q13 genes in both *SHANK3*-related and unrelated forms of PMS, provide valuable information to physicians about the expected head growth, and help early identification of brain abnormalities.

## 2. Materials and Methods

We reviewed the literature for 22q13 deletions published from 2000 to 2021, to identify cases with information on head circumference. PubMed and Google Scholar were searched using the terms (Phelan–McDermid syndrome OR 22q13) AND (macrocephaly OR microcephaly OR head size OR head circumference OR OFC OR growth). We excluded cases of 22q13 duplications or trisomy 22 because the goal of the study was to assess the effects of haploinsufficiency. For inclusion, deletions had to be defined either by terminal deletion size or breakpoint positions to identify the genomic region involved. Therefore, deletions of unknown size were excluded. All genomic coordinates were converted to genome build hg19 using the LiftOver tool (UCSC genome browser [10]). To determine whether a deletion preserved *SHANK3*, we used the coding region of *SHANK3* plus 1kb to account for promoter regions. Thus, we defined deletions ending at genomic position 51,112,070 (using genome build hg19) or more proximal on chromosome 22 as preserving *SHANK3*. We considered people carrying these interstitial deletions to be part of the subcategory of PMS *SHANK3*-unrelated [9].

Deletions were considered to include *SHANK3* if their proximal breakpoints were more terminal than this position. Cases due to *SHANK3* point mutations, rather than deletions, were not included because the extensive literature on *SHANK3* pathogenic variants encompasses not only PMS, but also autism spectrum disorder, intellectual disability, schizophrenia, and other neurodevelopmental disorders [11,12,13,14,15].

For this investigation, macrocephaly was defined as OFC of the 97th percentile or higher or reported as macrocephaly by the manuscript. Microcephaly was defined as an OFC of less than or equal to the 3rd percentile or reported as microcephaly in the manuscript. When OFC measurement was reported without a percentile, it was calculated from Simulconsult (https://simulconsult.com/, accessed on 9 March 2022) or using CDC growth charts. If values were reported as “>97”, they were coded as the 97th percentile for statistical analysis. Values “<3rd” were coded as the 3rd percentile for statistical analysis purposes. Finally, papers were required to be in English.

### Statistical Analysis

Data were analyzed using SAS v9.4. Descriptive statistics included the mean, median, standard deviation, and range. Comparisons of continuous variables including age, deletion size, and OFC percentile were assessed with the Wilcoxon Rank Sum test (2 group comparisons) and the Kruskal–Wallis test (3 or more group comparisons). Comparisons of categorical variables were assessed with Chi-Square or Fisher’s Exact test when expected cell sizes were *n* < 5. A *p*-value < 0.05 was considered statistically significant. Associations between segmental deletions and head size was conducted using PLINK v1.07 software [16] using 50,000 permutations to calculate empirical *p*-values with adjustment for multiple testing.

## 3. Results

A total of 56 published studies [5,8,11,14,17,18,19,20,21,22,23,24,25,26,27,28,29,30,31,32,33,34,35,36,37,38,39,40,41,42,43,44,45,46,47,48,49,50,51,52,53,54,55,56,57,58,59,60,61,62,63,64,65,66,67,68] were identified as having cases of 22q13 deletions along with information on the head size (either OFC measurement or classification as to macrocephaly, microcephaly, or normocephaly) (Table 1 and Table S1). The majority of publications (71%) were published since 2011, originated in Europe (61%) or North America (18%), and included individual case reports or small case series (83%). Descriptors that are included in Table S1 are author, year and countries of the studies; age, sex, and head circumferences of subjects; information about MRIs; deletion breakpoints; and size and *SHANK3* status. Shortened versions of Table S1 are Table 1 and Table 2.

A total of 198 individuals with 22q13 deletions were included in this pooled assessment (Table 2). Ages ranged from newborn to 70 years with a mean of 10.7 years (SD 12.3 years) and 53% of the samples were female. Almost all had deletion breakpoints provided in the manuscript while 14 (7%) provided terminal deletion sizes and thus breakpoints were estimated. Deletion sizes ranged from 12 kb to 9.4 Mb with a mean of 3.34 Mb (SD of 2.96). Approximately 18% had deletions smaller than 100 kb. Not all deletions were terminal; 25 deletions preserved *SHANK3* (13%), and 173 (87%) included *SHANK3* in the deletion. Head size was reported as “macrocephaly” for 17% of cases, “microcephaly” for 13% of cases, and “normocephaly” for the remaining 70% of cases. A subset of 108 subjects had information on OFC size and percentile. When the OFC percentile was available, 24% of cases were found to have macrocephaly, 18% were found to have microcephaly, and 58% were normocephalic. OFC percentiles ranged from 0.4 to >99.9 with a mean of 56.8 (SD 36.96). MRI information was available for 97 of the 198 subjects. A total of 73% percent of people with MRIs had abnormal findings such as atrophy, cysts (including arachnoid cysts in a variety of locations and choroid plexus cysts), abnormalities of the corpus callosum (including agenesis, thin, or atypical morphology), leukomalacia, hypomyelination, and abnormal ventricles.

### 3.1. Investigation of Factors Associated with Head Size

The median deletion size was larger for those with macrocephaly (5.32 Mb) than those with normocephaly (2.29 Mb) or microcephaly (2.20 Mb, KW *p* = 0.0310, Table 3). No differences in age (*p* = 0.5518) or sex (*p* = 0.9377) were noted for different head size classifications. Macrocephaly was more common among those with deletions preserving *SHANK3* (40%, 10/25) than among those with deletions including *SHANK3* (13%, 23/173), Fisher’s Exact (*p* = 0.0048) (Table 3). Microcephaly was present for 13% (22/173) of cases with *SHANK3* deleted and 16% (4/25) among those with *SHANK3* preserved (*p* = 0.8447, Fisher’s Exact). Similar patterns of larger deletion sizes were observed for those with macrocephaly among those with *SHANK3* preserved (median 4.15) or deleted (median 5.62 Mb), however, the differences were not statistically significant.

### 3.2. Assessment of Deletion Location on Macrocephaly, Microcephaly, and Normocephaly

**Figure 1:**
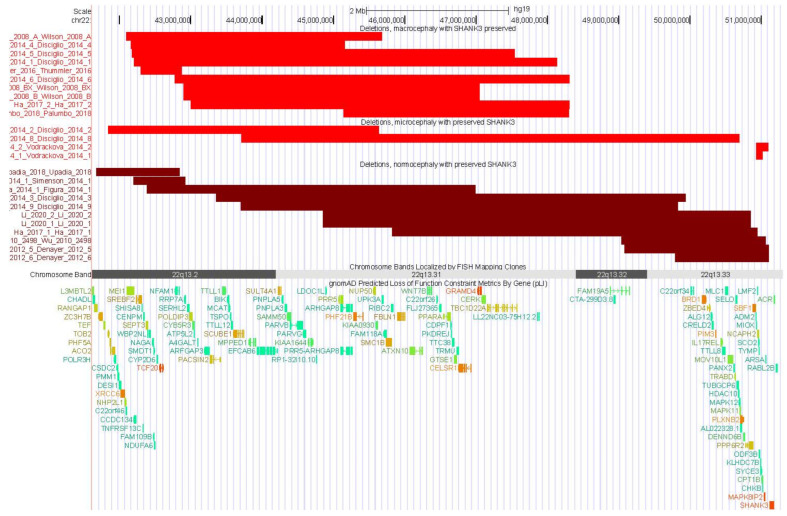
Distribution of deletions for individuals with macrocephaly (**top**), microcephaly (**middle**), and normocephaly (**lower**) with *SHANK3* preserved. The graphic demonstrates the preponderance of deletions in the 22q13.2 and 22q13.31 bands for those with macrocephaly whereas individuals with normocephaly have deletions across all of 22q13. No pattern of deletions is apparent for microcephaly among those with *SHANK3* preserved.

**Figure 2:**
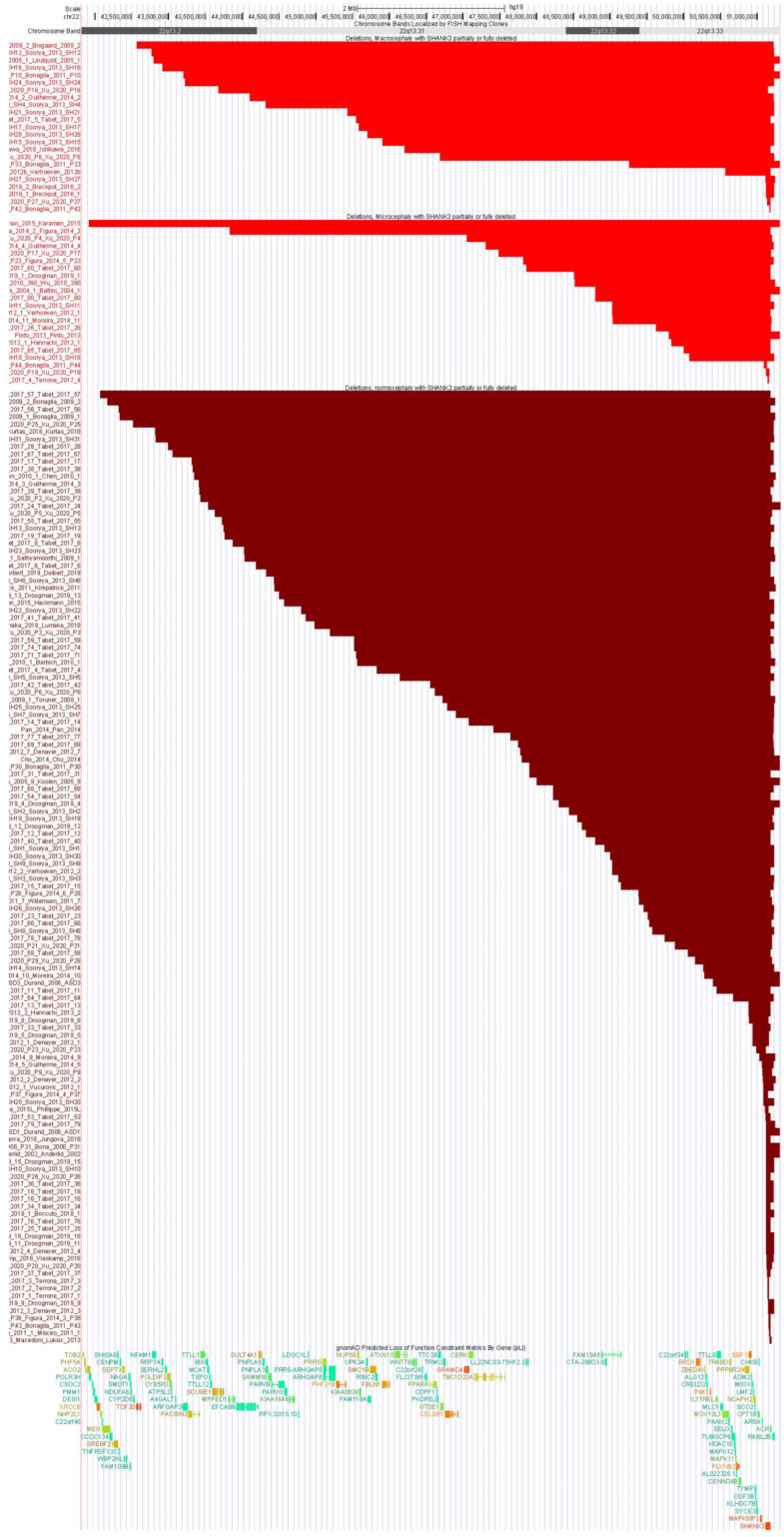
Distribution of deletions for individuals with macrocephaly (top), microcephaly (middle), and normocephaly (bottom panel) with *SHANK3* partially or completely deleted. Note the preponderance of deletions > 1 Mb among individuals with microcephaly compared to those with normocephaly. Note the preponderance of deletions > 4 Mb among those with macrocephaly.

Graphing the deletions for individuals with macrocephaly, microcephaly, and normocephaly for those with *SHANK3* preserved (Figure 1) and *SHANK3* not preserved (Figure 2) highlights that individuals with macrocephaly tended to have much larger deletions as well as deletions that began more proximally than subjects with normocephaly. Terminal deletions > 4 Mb in size showed the highest association with macrocephaly (Figure 2).

### 3.3. Association between OFC Measures and 22q13 Deletion Size

**Figure 3:**
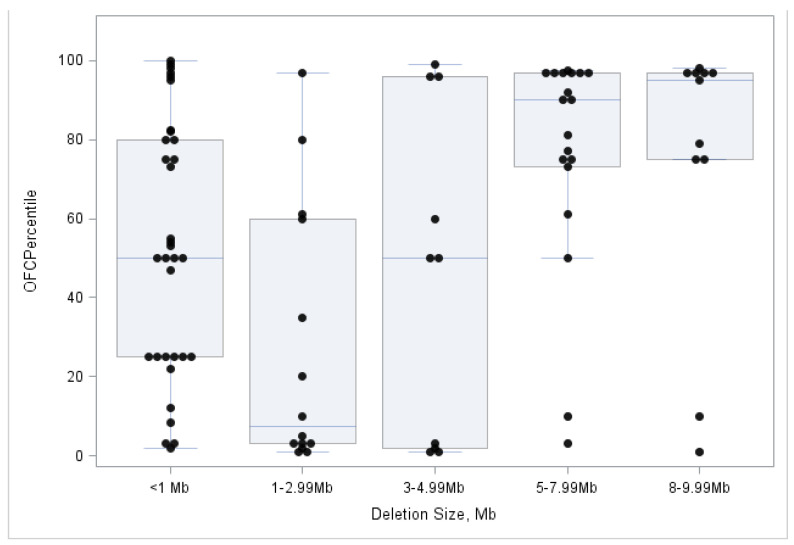
Distribution of OFC percentile by chromosome 22q13 deletion size for 87 individuals with partially or fully deleted *SHANK3*.

To perform a finer assessment of the association between deletions and OFC, we next examined the distribution of deletion size on head size among the 108 individuals with OFC percentile data. There was no difference in the OFC percentile for the 21 subjects with preserved *SHANK3* and the 87 with partially or fully deleted *SHANK3* (Wilcoxon Rank Sum *p* = 0.575). However, among the 87 subjects with partially or fully deleted *SHANK3* deletions, there was a significant association between deletion size and OFC percentile (Kruskal–Wallis *p* = 0.0016) (Figure 3). Individuals with larger deletions, particularly those with deletions > 5 Mb, were more likely to have larger heads.

### 3.4. Association between Head Size and MRI Results

A total of 97 (49%) individuals had information on MRI results. The majority (73%) of individuals with MRI data had an abnormality of some kind (Table 4). In general, head size was not associated with having an abnormal MRI, with the exception that microcephaly was associated with brain atrophy (*p* = 0.0272, Table 4).

### 3.5. Association between 22q13 Genomic Deletions and Macrocephaly

Given the association that was observed between deletion size and macrocephaly, an additional analysis was conducted to determine whether specific loci on chromosome 22q13 were associated with head size (Table 5). Association analysis between segmental deletions along 22q13 and macrocephaly compared to normocephaly identified two genomic regions that were significantly associated with macrocephaly, one in 22q13.2 with 14 genes was significantly associated for those with *SHANK3* preserved, and the other around 22q13.31 with 22 genes that was significantly associated for those with *SHANK3* deleted. For microcephaly, a region on 22q13.33 was nominally associated with microcephaly in comparison to normocephaly, but this region was not significant after correction for multiple testing. Following the criteria that were outlined by Mitz et al. (2020) to prioritize genes that were most likely to be haploinsufficient [69], three genes (TBC1D22A, CELSR1, and GRAMD4) are candidates as they are in the highly associated region, have a pLI score > 0.9, and a population impact factor of >0.5. Thus, approximately half of the people with Phelan–McDermid syndrome deletions of 22q13 are estimated to have deletions encompassing these genes. The high pLI score is a tool to help predict whether having only one functioning copy of these genes is estimated to be pathogenic, although it should be interpreted with caution [70].

## 4. Discussion

In this study of individuals with PMS due to 22q13 deletions, we found macrocephaly to be reported in 17% of subjects and microcephaly to be reported in 13%. When the OFC percentile was reported, the rates were 24% of subjects with macrocephaly and 18% of subjects with microcephaly. Macrocephaly was more common (40%) among those with preserved *SHANK3* (*SHANK3*-unrelated) than among those with *SHANK3* deleted (*SHANK3*-related group). We observed a strong association between larger head sizes and larger deletions, with a region in 22q13.31 containing 22 genes being the most associated. We did not observe an association between the reported head size and prevalence of specific MRI abnormalities, other than that brain atrophy was associated with microcephaly. This association is consistent with the hypothesis of prenatal pathogenesis of the brain atrophy in these subjects: the reduced brain volume affects the development of the skull resulting in microcephaly. Interestingly, other neuroanatomical abnormalities do not seem to influence the head size, suggesting that postnatal onset and/or their effect on skull development may not be relevant.

There are three genes that are proposed as the main candidates for macrocephaly (*TBC1D22A*, *CELSR1*, and *GRAMD4*) given their location in the highly associated genomic regions, proposed function, or high probability of loss of function intolerance [71]. *TBC1D22A* has been associated with structural or functional abnormalities of the head or the central nervous system (CNS), such as seizures, schizophrenia, or bipolar disorder [72,73]. Boucherie et al. (2018) found Celsr1-deficient mice to have microcephaly and cortical hypoplasia [74]. Nevado et al. (2022) identified a region of 4.5 to 8 Mb from the telomere as associated with macrocephaly and a region of 0.4 to 3.4 Mb from the telomere associated with microcephaly and suggest *GRAMD4* as a dosage-sensitive gene that may be involved in macrocephaly [3]. In particular, they point out that GRAMD4 interacts with the protein PIAS1, which has been associated with head size in the 19p13.3 deletion syndrome [3,75]. An association study suggested *WNT7B* to be a candidate gene for macrocephaly [76] although this gene is not predicted to be haploinsufficient according to the low pLI score of 0 [71]. We note that there are limitations regarding reliance on pLI scores [70], and therefore suggest *WNT7B* should be considered a candidate gene. If we consider all the 36 genes in the two regions that are associated with macrocephaly (14 on chromosome 22q13.2 and 22 on 22q13.31), it is of interest that biallelic pathogenic variants in the *RNU12* gene have been reported as causative of the CDAGS syndrome [77], characterized by craniosynostosis, delayed closure of the fontanelles, cranial defects, clavicular hypoplasia, anal and genitourinary malformations, and skin manifestations. The proven role of *RNU12* in cranial development and closure of the fontanelles suggests a potential double-hit pathogenic model for macrocephaly in subjects with PMS and loss of the chromosome 22q13.2 region, in which the deletion of one allele of *RNU12* is likely matched by a pathogenic variant on the preserved allele.

On the other end of the spectrum of abnormal head size, we observed a trend linking smaller 22q13 deletions to microcephaly, even if the association failed to reach statistical significance. This result is in line with the findings of a recent study by Malara et al. [78], indicating that deficiency of the SHANK3 protein in the post-synaptic density leads to myelin defects in the CNS. It is plausible that in subjects with smaller 22q13 deletions, the *SHANK3* haploinsufficiency may be responsible for the prevailing pathogenic mechanism influencing head development by reducing the levels of myelination and eventually leading to reduced brain volume and, indirectly, head size.

Our approach follows a similar methodology of pooling data to that which was performed by Samogy-Costa et al. [79], who combined data from four publications to assess associations between deletions and kidney disorders. Other approaches to examining large numbers of patients could include the use of registry data such as the PMSF International Registry [80] or prospective data collection on large cohorts [3].

### Limitations

This study was strengthened by the large sample size (198 people with genetic and head size data), spanning 56 unique publications across 22 countries. The large sample size allowed for investigations into associations between important features that are not possible from case reports or case series. The literature search was not exhaustive and may have missed some cases. Pooling of data across studies may have introduced errors. Some individuals may have appeared in multiple studies, but were only counted once for analysis and we did not observe identical deletions. The specific chromosomal aberrations causing the deletions (e.g., simple terminal deletion, ring chromosome, translocations, etc.) were inconsistently reported in the literature and not assessed in this analysis. Phenotypic data may have been reported in a non-systematic manner across the different studies, although OFC head size is a standardized measurement. This study relied upon the classification of head size by the respective authors and the actual OFC percentile was available for approximately half of the subjects. Half of the subjects had information on MRI, however, it is unknown whether the MRI data are representative of all individuals as there may have been specific indications for performing the test.

## 5. Conclusions

This study supports the suggestion that larger deletions, and most likely deletions of gene(s) located on 22q13.31, as associated with macrocephaly [3,73]. Future studies would benefit from a systematic collection of phenotypic data on OFC, MRI, and well a characterized genotype, including specific breakpoint positions and whole exome or genome sequencing to determine contributing variants elsewhere in the genome or on the preserved copy of 22q13. MRI reporting for persons with PMS could include the reason for obtaining the MRI, OFC at the time of the study, and genomic data.

## Figures and Tables

**Table 1:** Description of 56 published articles with information on genetic data and head size.

	Category	*n* (%)
Year of Publication	2000–2005 2006–2010 2011–2015 2016–2020	5 (9%) 11 (20%) 23 (41%) 17 (30%)
Country of Publication	Africa (DR Congo, Tunisia) Asia (China, Japan, Korea, Taiwan) Europe (Belgium, Czech Republic, Denmark, Estonia, France, Germany, Italy, Netherlands, Norway, Slovakia, Slovenia, Sweden, Turkey) North America (Canada, USA) South America (Brazil)	2 (4%) 7 (13%) 34 (61%) 10 (18%) 3 (5%)
Sample Size with Data	1 2–9 10–19 20–50	33 (59%) 19 (34%) 2 (4%) 2 (4%)

Citations: [5,8,10,13,17,18,19,20,21,22,23,24,25,26,27,28,29,30,31,32,33,34,35,36,37,38,39,40,41,42,43,44,45,46,47,48,49,50,51,52,53,54,55,56,57,58,59,60,61,62,63,64,65,66,67,68].

**Table 2:** Description of 198 cases in the literature with 22q13 deletions with complete data on deletion size and head size.

Variable	*n* = 198
Sex Male Female	93 (47%) 105 (53%)
Age of OFC measurement: Mean (SD)	10.7 years (12.3)
Age of OFC measurement: *n* (%) Newborn—4 years 5–11 years 12–17 years 18–70 years	82 (41%) 60 (30%) 20 (10%) 36 (18%)
Size of Deletion: mean (SD)	3.34 Mb (2.96)
Size of Deletion: *n* (%) <100 kb 100–999 kb 1–2.99 Mb 3–4.99 Mb 5–6.99 Mb 7–9.4 Mb	35 (18%) 28 (14%) 41 (21%) 29 (15%) 32 (16%) 33 (17%)
*SHANK3* preserved: *n* (%) Yes No	25 (13%) 173 (87%)
Mosaic: *n* (%) Yes No/unknown	5 (3%) 193 (97%)
Inheritance: *n* (%) Maternally inherited Paternally inherited Inherited/unspecified *De novo* Unknown	4 (2%) 6 (3%) 4 (4%) 115 (59%) 69 (35%)
Head size classification: *n* (%) Macrocephaly Normocephaly Microcephaly	33 (17%) 139 (70%) 26 (13%)
OFC Percentile when reported: mean (SD)	56.8 (SD 36.96)
OFC Percentile when reported: *n* (%) ≥97th 4th–96th ≤3rd missing	26 (24%) 63 (58%) 19 (18%) 90 (--)
MRI Performed: *n* (%) Yes No	97 (49%) 101 (51%)
MRI findings Normal Abnormal Type of abnormality * Atrophy Corpus callosum abnormalities Enlarged/abnormal ventricles Leukomalacia Cysts Hypomyelination Other	26 (27%) 71 (73%) 12 (12%) 22 (22%) 21 (22%) 20 (21%) 12 (12%) 11 (11%) 11 (11%)

* Type of MRI abnormal finding adds to more than 100% as more than one finding could be reported on an abnormal MRI. OFC occipitofrontal circumference (head circumference)

**Table 3:** Differences in 22q13 deletions and demographics by the size of the head.

	Macrocephaly	Normocephaly	Microcephaly	*Kruskal Wallis* *p*-Value
*n*	*n* = 33	*n* = 139	*n* = 26	
Median deletion size, Mb (hg19)	5.32 Mb	2.29 Mb	2.20 Mb	**0.0310**
Median age	6.0	5.25	6.0	0.5518
% Male	45%	50%	47%	0.9377
***SHANK3* partially or fully deleted**	*n* = 23	*n* = 128	*n* = 22	
Median deletion size, Mb ± SD (hg19)	5.62 (3.25)	2.17 (3.07)	2.29 (2.22)	0.0788
Median age in years (SD)	7.0 (18.8)	5.9 (11.6)	5.0 (7.5)	0.3776
***SHANK3* preserved**	*n* = 10	*n* = 11	*n* = 4	
Median deletion size, Mb ± SD (hg19)	4.15 (1.61)	4.60 (2.33)	1.99 (3.30)	0.6696
Median age in years (SD)	5.5 (6.89)	7.0 (14.4)	8.25 (6.25)	0.7848

**Table 4:** Differences in 22q13 deletions and MRI findings by head size.

	Macrocephaly	Microcephaly	Normocephaly	Fisher’s Exact *p*-Value
MRI (*n* = 97) Normal Abnormal	3 (20%) 12 (80%)	2 (14%) 12 (86%)	21 (31%) 47 (69%)	0.4322
Brain Atrophy Yes No	1 (7%) 14 (93%)	5 (36%) 9 (64%)	6 (9%) 62 (91%)	**0.0272**
Corpus Callosum ab. Yes No	4 (27 %11 (73%)	5 (36%) 9 (64%)	13 (19%) 55 (81%)	0.3354
Cysts Yes No	3 (20%) 12 (80%)	0 (0%) 14 (100%)	9 (13%) 68 (87%)	0.2761
Hypomyelination Yes No	1 (7%) 14 (93%	1 (7%) 13 (93%)	9 (13%) 59 (87%)	0.8908
Leukomalacia Yes No	5 (33%) 10 (67%)	2 (14%) 12 (86%)	13 (19%) 55 (81%)	0.4594
Enlarged/Abnormal ventricles Yes No	4 (27%) 11 (73%)	4 (29%) 10 (71%)	13 (19%) 55 (81%)	0.5468
Other Abnormality Yes No	2 (13%) 13 (87%)	2 (14%) 12 (86%)	7 (10%) 61 (90%)	0.6853

Bold font indicates statistically significant.

**Table 5:** Genomic regions (hg19) and genes that are associated with macrocephaly or microcephaly as compared to normocephaly. Corrected for multiple testing using the Benjamini–Hochberg method as implemented in Plink software.

Head Size	Genomic Region Nominally Associated *p* < 0.05	The Genomic Region Associated after Benjamini–Hochberg Correction (*p* < 0.05)	Genes Significant in Regional Tests after Benjamini–Hochberg Correction, *p* < 0.05
Macrocephaly, *SHANK3* preserved	42.90–44.85	42.92–43.36	14 genes: *SERHL2, RRP7B, POLDIP3, RNU12, CYB5R3, ATP5L2, A4GALT, ARFGAP3, PACSIN2, TTLL1, BIK, MCAT, TSPO, TTLL12*
Microcephaly, *SHANK3* preserved	None	None	None
Macrocephaly, *SHANK3* partially or fully deleted	45.58–49.00	46.69–46.91	22 genes: *ATXN10, WNT7B, LOC730668, LINC00899, PRR34, LOC150381, MIRLET7BHG, MIR3619, MIRLET7A3, MIR4763, MIRLET7B, PPARA, CDPF1, PKDREJ, TTC38, GTSE1-AS1, GTSE1, TRMU, CELSR1, GRAMD4, CERK, TBC1D22A*
Microcephaly, *SHANK3* partially or fully deleted	50.00–50.94	None	None

## Data Availability

No new data were generated in this investigation. The data abstracted from the literature is provided as Supplementary Material.

## Supplementary Materials

The following supporting information can be downloaded at: https://www.mdpi.com/article/10.3390/genes14030540/s1, Table S1: Head size in PMS; literature data abstraction.xlsx.
